# 

**Published:** 1849-03

**Authors:** 


### CONTENTS

OF NO. III.

---

### ORIGINAL COMMUNICATIONS.

### ESSAYS AND CASES.

ART. I.—Tyhhoid Fever as it appeared in the vicinity of Heltonsville during the winters of 1846–'7–'8. By Dr. M. H. FEE, of Heltonsville, Indiana....185

ART. II.—Cases of Acute Articular Rheumatism, treated in Blockley Hospital. By B. F. WENDEL, M.D., Assistant Pnysician....193

ART. III.—A brief account of a Fever which prevailed in the mountain district of Tennessee, in the Fall and Winter of 1847–'8. By Dr. M. Y. BROCKETT, of Spencer, Tennessee....199

ART. IV.—A Case of Meningo-Cerebritis. By Dr. THOMAS C. OSBORNE, of Erie, Alabama....202

ART. V.—Sudden Development of Anomalous Tumors. By JOHN A. COTTON, M.D., of Greenwood, Miss....204

ART. VI.—Notice of the Galium Tinctorum as a Remedy in Chronic Diarrhea. In a letter to the Editor, by Dr. THOMAS C. OSBORNE, of Erie, Alabama....206

ART. VII.—Comparative Mortality of Typhoid Fever in Kentucky and Missouri. In a letter to the Editor, by S. W. SOUTHWORTH, M.D., of Glasgow, Missouri....208

ART. VIII.—A Case of Pleuro-Pneumonia of the right Lung; Bronchitis of the left, complicated with Inflammation of the Liver, followed by fatal Pericarditis. Autopsy. By DAVID W. YANDELL, M.D., of Louisville, Ky....209

### REVIEWS.

ART. IX.—A Theoretical and Practical Treatise on Human Parturition. By H. MILLER, M.D., Professor of Obstetrics and Diseases of Women and Children, in the Medical Department of the University of Louisville....219

SELECTIONS FROM AMERICAN AND FOREIGN JOURNALS.

Cholera as it prevailed in New Orleans......................245
On the Treatment of Remittent and Intermittent Fever by Quinine in the West Indies ..............................253
Muriate of Opium..........................................258
Death from Chloroform.....................................258
Collodion and Asbestos in Toothache.......................259
Presence of Sugar in the Liver............................260
Dr. Sutherland on the Treatment of Cholera ...............260
Course of Cholera.........................................262
On the utility of Alkalies in the Treatment of Rheumatism......266
Case of Poisoning by Arsenic .............................267

ORIGINAL INTELLIGENCE.

Cholera ..................................................269
Cholera in Nashville—Relation to Limestone................271
Tone of the Brain Improved by Disease ....................272
Editorial Changes ........................................274
Personal Portraits .......................................275
Death of Mr. Samuel Cooper ...............................276

